# Assessment of communication skills using telehealth: considerations for educators

**DOI:** 10.3389/fmed.2022.841309

**Published:** 2022-08-01

**Authors:** Hattie H. Wright, Marie-Claire O’Shea, Julia Sekula, Lana J. Mitchell

**Affiliations:** ^1^School of Health and Behavioural Sciences, University of the Sunshine Coast, Maroochydore, QLD, Australia; ^2^Sunshine Coast Health Institute, Britinya, QLD, Australia; ^3^School of Health Sciences and Social Work, Griffith University, Southport, QLD, Australia; ^4^Faculty of Medical and Health Sciences, The University of Auckland, Auckland, New Zealand; ^5^Menzies Health Institute Queensland, Southport, QLD, Australia

**Keywords:** communication, dietitian, education, simulation, student, telehealth, telemedicine, university

## Abstract

**Objective:**

The main aim of this study was to explore the views and perceptions of dietetic educators on their ability to assess communication skills of undergraduate student dietitians in a telehealth setting. A secondary aim was to provide recommendations to educators when assessing these skills using telehealth.

**Methods:**

A descriptive qualitative study design was used. Australian and New-Zealand dietetic educators used a validated global communication rating scale to evaluate three pre-recorded telehealth encounters. Educators then answered a series of open-ended questions on their ability to assessed communication skills in the telehealth environment.

**Analysis:**

Inductive analysis allowed the emergence of themes and sub-themes independent of a specific framework or theory. Peer debriefing and triangulation increased research rigor.

**Results:**

Twenty-four educators were included in this study with the majority (87.5%) having > 10 years experience as a dietetic educator, and 41.6% (*n* = 10) with experience in assessing dietetics student using telehealth. Most (76%) educators reported the assessment of non-verbal communication skills were challenging in the telehealth environment. Five themes and 15 subthemes emerged relating to advice for students and educators when assessing communication skills and a checklist was developed from recommendations that students and educators can use when preparing, planning, implementing, and assessing telehealth consultations.

**Conclusion:**

Assessing student communication skills *via* telehealth provides a useful opportunity with the growing use of the online environment, however, it also presents challenges that must be taken into consideration. While verbal communication skills are easier to assess than non-verbal, both need to be adapted for the telehealth setting.

## Introduction

In the wake of the COVID-19 pandemic the education sector had to rapidly shift to an online learning environment. This posed unique challenges and opportunities for healthcare education, assessment, and skill development. Telehealth is one such opportunity that was embraced by universities, clinicians, and students alike (1–4). Telehealth is defined as the use of information and communication technology for persons and communities who have difficulty in accessing their healthcare provider (5). Telehealth provides telemedicine, medical education, and health education without the constraints of distance. It is seen as the future of healthcare as it enables access to quality healthcare for those that are constraint by time and distance (5–7).

Telehealth has shown promise to help overcome patient-centered and health care barriers to health management (8), and within dietetic service delivery to be effective in the provision of nutrition interventions including malnutrition (9), and management of non-communicable disease (10–12). However, prior to the COVID-19 pandemic, telehealth was not necessarily incorporated into the curricula of dietetics and other healthcare programs or widely used in service delivery due to various concerns and challenges (i.e., quality of care, ethical, and legal issues) (13). Nevertheless, evidence emerged on the potential benefit of using telehealth as an alternative setting for student skill development (14, 15), with an e-Health skills component positively received by dietetic students (16). In 2020, despite limited time, resources and readiness to embed telehealth into dietetic curricula, educators had to quickly adapt in response to the unfolding pandemic and utilize telehealth as an alternative setting for student training prior to and during clinical placement (3). With the surge in the uptake of telehealth, professional bodies developed guidelines to support dietitians in the implementation of telehealth (17, 18). Recently a United States-based study surveyed registered dietitians about their perceptions of telehealth in practice prior to and during the pandemic. The study identified several opportunities afforded by telehealth including longer assessment times with patients and enabling an insight into the home environment, not otherwise seen in face-to-face consultations (19). Similarly, Mehta and colleagues (20) identified several positive aspects to telehealth and concluded that telehealth is likely to remain as a component to dietetic practice. This highlights the importance for dietetics educators to effectively implement and design experiential learning opportunities which incorporates telehealth into curricula in order to best equip future dietitians in the delivery of healthcare now and post-pandemic. Currently there is paucity in resources and guidance on the assessment of dietetic skills in a telehealth setting.

An area of concern in the implementation of telehealth is the risk of depersonalization in the clinician-patient relationship due to the lack of human contact and ability to pick up on non-verbal cues (13). The importance of appropriate interpersonal skills for the telehealth setting has also been highlighted by Henry et al., as well as the recognition that non-verbal communication is difficult to facilitate in the online environment (21). Good communication skills are a key attribute of health care professionals and is central to the delivery of optimal health care (22). In fact, effective communication skills positively impact overall health outcomes and compliance (23, 24), improve patient satisfaction (25), and self-management (26), and has been linked with reduced malpractice claims (27). In dietetics, increased patient satisfaction was found with dietetic consultations where dietitians display effective non-verbal skills, was able to build rapport, and showed empathy (25, 28, 29). It is therefore not surprising for the growing need in development and skill acquisition of verbal and non-verbal communication skills of student dietitians (29). The importance of communication skills is further acknowledged and reflected in the professional competency standards for dietitians in both Australia (30) and New Zealand (31). These state that dietitians should display effective communication and interpersonal skills that establish and maintain professional relationships using a client-centered approach.

Given the importance of communication skills in health care delivery, the challenges of communication in the telehealth setting, and the lack of best practice guidelines in the training and assessment of communication skills in the online environment, further research is needed to understand the unique challenges faced by educators when assessing communication skills in an online setting. The study aim was to explore the views and perceptions of dietetic educators on the assessment of communication skills of undergraduate student dietitians using telehealth and seeks to provide educators with recommendations for assessment of these skills *via* telehealth. The following research questions were therefore posed: What are the views and perceptions of dietetic educators on their ability to assess communication skills of students delivering nutrition care *via* online video conferencing? How can dietetic educators prepare themselves and their students for telehealth delivered *via* online video conferencing?

## Materials and methods

### Study design and sampling

A descriptive qualitative study design explored the views and perceptions of dietetic educators on their ability to assess communication skills using telehealth. A qualitative description design was selected as this methodology seeks information directly from those with the shared experience of the phenomenon (32).

Purposive sampling was conducted of Accredited Practicing Dietitians (APDs) in Australia and Registered Dietitians (RDs) in New Zealand with experience in the skills assessment of pre-placement dietetics students. Recruitment occurred through an established Australian-New Zealand community of practice of dietetics educators as well as through the research teams’ various university and placement partner networks. Participants were asked to view telehealth dietetic interview recordings and assess the communication skills of pre-placement dietetics students using a previously validated global rating scale from medicine (33). The aim of this study was not to evaluate the communication skills of students thus scores are not reported here.

Three simulated telehealth video recordings of different dietetics students that successfully passed a communication and counseling practical exam were selected for this study. Passing students were chosen to allow the participants to focus on the telehealth platform rather than student quality, in line with the study focus. The practical exam was part of a third-year capstone course prior to clinical placement. The communication and counseling practical exam followed the Nutrition Care Process (34). Students were given 40 min to prepare for their consultation under exam conditions using a referral letter and food diary. They then undertook a 20-min online consultation with a simulated patient focusing on clarification of data assessment information, nutrition education and nutrition counseling (goal setting and negotiation of strategies), as per the Nutrition Care Process (34). Two researchers (blinded for peer review) identified the most suitable recording to represent each distinct part of the interview to be used for this study. Selection was based on the potential for students to display relevant communication skills, as assessed by the global rating scale (33), which would afford assessors to comment on their ability to assess communication skills through telehealth. Simulated consultations were conducting and recorded using the online videoconferencing system Microsoft Teams.

Ethical approval was gained from the Research and Ethics committee of [Griffith University] (No. 2020/881). Students and simulated patients provided written informed consent to be recorded and for the recordings to be used for future training and research purposes.

### Data collection and analysis

Demographic information was gathered through an online survey and included primary workplace, years of practice, years of experience as an assessor of pre-placement skills, and previous assessment of dietetic skills *via* telehealth.

#### Participant views and perceptions

After participants watched the video recordings and scored students’ communication skills, they were asked to reflect on their ability to assess communication skills *via* a telehealth recording through answering a series of open-ended questions using an online written survey: “*What communication factors do you believe were easily assessed? Where possible, please provide an explanation*,” “*What communication factors do you believe were not able to be adequately captured? Where possible, please provide an explanation*,” “B*ased on your experience, in what ways could this marking experience be improved?*,” “*What advice do you have for university educators setting up telehealth consultations for assessing student readiness for placement?*,” and “*What advice do you have for students to improve communication skills when consulting via telehealth?*”

#### Analysis

Participant characteristics are reported as frequencies and percentage of the total group. Open-ended questions were analyzed using qualitative description which allowed inductive thematic analysis which is not guided by a theory or framework and thus allows the opportunity for new or unexpected understandings (35). Thematic analyses were conducted according to the six-phase process of Braun and Clarke (36). Responses to open-ended questions were organized by descriptive coding into topics reading through each response and organizing into subthemes. Coding was done in duplicate by “HW” and “MCO” (dietetic educators with extensive dietetic education and simulation-based learning experience, including telehealth experience) and reviewed by “JK” (5 years’ experience in simulation-based learning and extensive dietetic education experience). Subthemes were then grouped into overarching themes by “LM and HW (LM has extensive dietetic education experience) (37). To reduce risk of subjective bias peer debriefing was used during the finalizing of subthemes and themes with all members of the research team involved in the process. To gain insight into the agreement of educators on the ease of assessment of non-verbal and verbal communication factors inductive content analysis was also performed and is presented as frequency of agreement (38). A checklist was developed by the researchers for educators who assess communication skills using telehealth. Checklist items were identified from study results through a review of comments across all questions by the researchers.

## Results

Twenty-four participants were recruited, representing New Zealand and five Australian states. Participant characteristics are outlined in Table 1, with individual participant details in Supplementary Material. The majority of participants were working in the university setting (88%) with 10 or more years in the workforce (88%) and most had past experience assessing dietetic students using telehealth (63%).

**TABLE 1:** Demographic information and characteristics of participants.

	Frequency (*n* = 24)	Percentage total group (%)
**Primary workplace**		
University	21	87.5
Hospital	2	8.3
Private practice	1	4.2
**Dietetic registration**		
Accredited practicing dietitian (APD)	13	54.2
Registered dietitian (RD)	9	37.5
Both APD and RD	2	8.3
**Country, state**		
Australia	13	54.2
New South Wales	4	16.7
Northern Territory	0	0.0
Queensland	4	16.7
South Australia	1	4.2
Victoria	3	12.5
Western Australia	1	4.2
New Zealand	11	45.8
**Dietetic experience**		
1 – < 3 years	1	4.2
3 – < 5 years	1	4.2
5 – < 7 years	0	0.0
7 – < 10 years	1	4.2
10 – < 15 years	9	37.5
15 – < 20 years	5	20.8
20 + years	7	29.2
**Experience assessing dietetic students prior to clinical placement**		
2 years or less	3	12.5
3 –5 years	4	16.7
>5 years	17	70.8
**Experience assessing dietetics students using telehealth**		
No experience	9	37.5
Yes, prior to practical placement	7	29.2
Yes, prior to and during practical placement	3	12.5
Yes, during practical placement	5	20.8

### Communication factors assessable using telehealth

A summary of participants’ reported ease of assessment of different communication factors using telehealth is summarized in Table 2. Ten participants (40%) indicated that verbal communication skills were easy to assess. Specific reference was made to “tone and volume,” “voice change,” “paraphrasing,” “verbal cues,” “minimal encourages,” “verbal followings,” “summarizes” and clear audio. As one educator reported: “*it is easier to assess most verbal communication skills*…” (P9).

**TABLE 2:** Categorization of qualitative comments about ease of assessment of communication factors using telehealth, *n* = 24.

Communication factor reported	Easily assessed *n* (%)	Same as in person *n* (%)	Harder to assess *n* (%)
All skills, both verbal, and non-verbal communication	7 (29.2)	1 (4.2)	–
Logical flow and structure of interview	2 (8.3)	1 (4.2)	–
**Non-verbal**			
Reference to general non-verbal communication	–	–	6 (25.0)
Eye contact	–	–	5 (20.8)
Body language	–	–	7 (29.2)
Gestures	–	–	1 (4.2)
Empathy	3 (12.5)	1 (4.2)	–
Cues/Expressions	–	–	4 (16.7)
Rapport	1 (4.2)	–	3 (12.5)
Posture	–	–	2 (8.3)
Use of pause	–	–	1 (4.2)
**Verbal**			
Reference to general verbal communication	6 (25.0)	–	–
Cues/expressions	1 (4.2)	–	1 (4.2)
Tone and volume	1 (4.2)	–	–
Voice change	1 (4.2)	–	–
Counseling skills	1 (4.2)	–	–

Seven (28%) participants indicated “… *it was easy to assess all communication factors”* (P5) and “*verbal expression and non-verbal expression are easily seen on video”* (P8). The fact that the encounter was recorded was viewed as beneficial to participants, for example: “… *[you] can replay sections to review and watch the student vs. the patient”* (P5). The structure of the interview process and empathy was viewed easy to assess and “… *similar to any session whether telehealth or face-to-face counseling”* (P20).

The majority of participants (*n* = 19, 76%) reported non-verbal communication “… *is somewhat challenging to assess”* (P16), particularly factors such as “*eye contact and body language*…” (P12). An unclear view of the student and patient’s body made it hard to assess some elements, for example: “… *body language of the student and also ability of the student to pick up on client cues as [the assessor] can only see the patient’s head and neck and upper body of the student. Eye contact is [also] difficult via telehealth as [students] tend to look at the client on the screen rather than the camera.”* (P22). Some participants found it challenging to pick up on the client’s non-verbal cues which made it hard for assessors to judge “… *whether patients are engaged with the consult or not”* (P10). Rapport building was identified as “… *often more difficult using telehealth*…” (P6) and needs to be considered when assessing a student. Difficulty assessing students’ posture and some gestures were also mentioned. Despite the challenges identified to assess non-verbal communication factors using telehealth, assessors reported that it was “*not impossible”* (P3) but “…*more difficult than verbal skills”* (P11).

There were contrasting views on the mode of assessment with the feeling that “… *telehealth was not dissimilar to face-to-face methods”* (P21) compared to “… *assessment can only be in relation to telehealth, not another situation such as face-to-face, because the students may have used these skills differently in a face-to-face communication*…” (P20).

### Advice to educators and students on communication skills when consulting *via* telehealth

Participants were asked to provide advice to dietetic educators when setting up telehealth consultations and assessing recorded telehealth consultations, as well as to student dietitians on how to improve their communication skills when using telehealth. As there was consistent overlap between advice for students and dietetic educators these questions were analyzed together. Overall, five key themes and 15 subthemes were identified, as summarized in Table 3.

**TABLE 3:** Themes and subthemes identified from advice to students and dietetic educators to improve communication skills when consulting *via* telehealth.

Theme	Subtheme
1. Non-verbal communication is more difficult using telehealth	1.1 Non-verbal communication is important
	1.2 Focus on upper body gestures, in particular eye contact
2. Adapt verbal communication for telehealth	2.1 Use strategies to facilitate engagement
	2.2 Rapport building is more difficult online
	2.3 Ensure speech is clear with pauses to not speak over clients
	2.4 Continue to focus on elements that are not unique to telehealth
3.Establish an optimal telehealth environment	3.1 Ensure technology is working
	3.2 Create an ideal environment with minimal distractions
	3.3 Use online tools to maximize communication
4. Telehealth specific preparation	4.1 Provide sufficient training for students in telehealth
	4.2 Practice using telehealth
	4.3 Consider ways to provide real-time feedback and opportunities for students to reflect on performance to improve practice
5. Assessing *via* telehealth	5.1 Beneficial setting to assess and complements in person assessment
	5.2 Logistics to ensure assessable telehealth environment
	5.3 Standardized and clear assessment tools are required

#### Theme 1. Non-verbal communication is more difficult using telehealth

Participants highlighted that awareness of non-verbal communication is particularly important (Subtheme 1.1) in the telehealth environment. Students need to be made aware of their own non-verbal cues as well as those of the patient and the importance of adapting non-verbal communication for the telehealth setting. Dietetic educators can encourage students to focus on non-verbal communication by “*Reiterating the importance of*…. *non-verbal cues”* (P2) and provide suggestions how to optimized their non-verbal cues:

“*The client can still see you so your non-verbal communication skills from the waist up [is] very important.”* (P1)

Participants reported the telehealth environment to pose a challenge for students to demonstrate their non-verbal communication skills, as well as educators to assess these skills. As illustrated by these participants:

“… *non-verbal communication is more difficult to demonstrate via telehealth.”* (P17)

“*Some ‘leniency’ is required around assessing body language and similarly non-verbal patient cues as these can be lost via telehealth.”* (P22)

Telehealth can often limit visibility of the whole body, therefore it is important to focus on upper body gestures (Subtheme 1.2) and maintain eye contact “*even if it feels artificial”* (P12). The camera set-up of the student and the location of the patient’s image on their screen can reduce the ability of the student to monitor or assess non-verbal cues, therefore students need to be reminded to “*look at the camera*” (P22) so that the patient has a clear view of the student’s face and the student appears engaged. Students need to be reminded to adapt their non-verbal communication for the telehealth environment, as too much movement can be distracting:

“*Be careful with hand gestures as these can be really distracting on a small screen*.” (P24)

#### Theme 2. Adapt verbal communication for telehealth

Subtheme 2.1 highlights the requirement to use strategies to facilitate engagement, despite being in a different location so the patient does not feel distant and become disengaged:

“…[the] i*mportance of making effort to engage with patient even though not in same room.”* (P17)

Use of active listening skills such as “*reflecting back”* (P19), “*screen sharing”* (P12) to help with education, and provide space (for example, pausing) to allow patients time to ask questions and engage was mentioned:

“*Regularly check in with a quiet patient as it can be more difficult for a patient to interrupt via telehealth.”* (P22)

Verbal communication needs to be adapted, sometimes to make up for limitations in non-verbal cues and other times needs to be cut back:

“… *strategies [is needed] to counteract non-verbal body language potentially being diminished during video consults.”* (P19)

“… *to minimize patient interruptions with yip and respond in non-verbal ways so that the clients flow is not broken.”* (P1)

Due to the nature of telehealth, students may be tempted to follow a pre-determined script during their consult. This limits engagement with the patient, with responses less likely to be tailored to the patient’s needs:

“*Check students are not just reading from a (hidden) script but adapting the consultation to the patient.”* (P7)

It was also identified that rapport building is more difficult online (Subtheme 2.2), as highlighted here:

“*To develop rapport online can be more difficult for some so make sure you cover what the client wants and what they value.”* (P1)

Subtheme 2.3 highlighted the need to ensure speech is clear with pauses to not speak over clients and the pace is not too fast. This is important so clients can navigate an unfamiliar setting, manage “*transmission delays”* (P19), as well as provide sufficient time for clients to speak:

*“It is important to not speak over patients, it is harder with telehealth to sometimes identify the pauses, so always telling students it is okay to have some pauses in conversations to allow patients to speak.” (P9)*

Participants also recommended continuing to focus on elements that are not unique to telehealth (Subtheme 2.4). The opportunity to observe experienced clinicians or have “*recorded examples”* (P16) will assist in learning:

“*Like any situation, I think telehealth requires observation of a clinician who does it well (or preferably more than one to observe different styles).”* (P20)

Other similarities such as maintaining a clear consultation “*structure”* (P2) and flow, utilizing effective “*counseling skills”* (P14), assess patient receptiveness and “*readiness to change”* (P11). The importance of active listening and clarifying patient understanding were also highlighted:

“*Incredibly important not to assume patient understands what is being said.*” (P2)

The importance of student “*reflection post consult”* (P14) and the opportunity for debriefing with the client after the interaction was recognized to extend and deepen the learning experience: “*client feedback perhaps re how they felt.”* (P3)

#### Theme 3. Establish an optimal telehealth environment

A key factor to optimizing the telehealth environment was to ensure the technology is working (Subtheme 3.1). This included internet connection, clear sound and visuals so the client can “*hear and see before commencing”* (P18). Dietetic educators were encouraged to provide technical support to students during consultations, oversee general tasks such as ensuring the client is comfortable and the session is being recorded if required:

“*Ensure the technology is working and the record button is clicked!”* (P7).

Creating an ideal environment with minimal distractions (Subtheme 3.2) was emphasized. Students were encouraged to “*Be organized so not shuffling lots of papers*” (P7) as well as be aware of their surroundings. A quiet space with minimal background noise, appropriate lightning and minimizing distractions was recommended:

“*Explaining to them how distracting small things can be (like fan behind student’s head*…, *moving hands etc.*)” (P2).

Educators should make use of a “*waiting room”* (P12) functionality to ensure confidentiality and minimize distractions whilst in consultation. When using telehealth for assessment, participants highlighted the benefit of “*Getting good ‘[simulated] patients”’* (P23).

Finally, in order to establish an optimal telehealth environment, participants recommended the use online tools to maximize communication (Subtheme 3.3). Educators and students should familiarize themselves with the functions of the videoconferencing platform used in order to create an environment that supports communication and engage the client. Utilizing the online whiteboard, screen sharing and highlighting functions to facilitate education were recommended:

“*It is important to use the functions to share the screen and show resources to aid patient understanding, don’t just talk as people get distracted”* (P9).

In addition, the benefit of emailing education resources and a written plan to the patient following the consultation was identified: “*encourage them to have a written plan that they could email to the patient*” (P2).

#### Theme 4. Telehealth specific preparation

Sufficient training for students in telehealth (Subtheme 4.1) is required. Participants felt checklists and tip sheets on verbal and non-verbal communication skills will support student training. Specific elements to include in training are setting up the telehealth consultation, confidentiality, patient engagement, and differences in verbal and non-verbal communication:

*“At our Uni, I feel like we have transitioned to telehealth quickly/everything was done on the fly because we had to and now need to go back and do some work with students around how to best set up telehealth consults, how communication might differ/similarities and differences, additional privacy”* (P16)

Subtheme 4.2 then highlights the need for students to practice using telehealth either in class with peers or formal simulations is required “*to develop and fine tune the specific skills required for [telehealth] context*” (P20). The importance of students practicing individually was also highlighted:

“*Practice in front of a camera to review self-awareness including movements which could be distracting*” (P5).

Educators were encouraged to consider ways to provide real-time feedback and opportunities for students to reflect on performance to improve practice (Subtheme 4.3). Telehealth provides the ability for timely feedback as well as coaching by using the chat function. Recording consultations can afford students the opportunity to replay, reflect and improve their skill development but also provide educators the opportunity to provide further feedback:

“*I’ve found that tele-health provides good opportunity for coaching during the consultation using the chat functions and this has been valuable”* (P12).

#### Theme 5. Assessing *via* telehealth (dietetic educators only)

Participants clearly identified that telehealth was a beneficial setting to assess that complements in person assessment (Subtheme 5.1). Teaching students to be adaptable and flexible in translating their skills to different settings within an “*ever-changing health system”* (P1) was voiced. Telehealth is a growing area for delivery of healthcare and participants identified the value of including telehealth training and assessment in the curriculum:

“*I think the telehealth consultation is important to include, as it is very relevant to the current climate and very likely would continue to be relevant to practice moving forward.”* (P20).

Telehealth does present multiple benefits for assessment, including its flexible nature and ability for assessors to be present but unseen.

*“The fact that the assessor’s face cannot be seen during the interview can be quite empowering for the student—patients cannot look to supervisor for input and the student is allowed to ‘own the space,’ whilst still having the opportunity to call the supervisor into the ‘room’ if needed.”* (P24)

Some participants identified the benefit of using telehealth in addition to face-to-face:

*“A mix of both telehealth and face-to-face consultations would be important as these different mediums could have different effects on student’s confidence and building communication skills using both mediums are important skills to have in today’s environment.”* (P18)

The additional complexity that telehealth brings to skill development and assessment compared to a face-to-face setting was acknowledged:

*“There is just the added complexity of working with the students to develop their dietetic skills and then also work with them to develop the technology skills needed for telehealth assessments*… *how to show resources and explain DDR [diet-disease relationship], how to check patient understanding*… *and pick up on cues etc.”* (P9)

There were a variety of logistics to ensure assessable telehealth environment (Subtheme 5.2) that need to be considered. When organizing cases and simulated patients it is important to have all the required details set up at the beginning, including: “*Pre-arranged consent”* (P14): and “*adequate patient information re background, medical status etc.”* (P17): “*Make sure you have your cases well set up with a very simple referral for the student and much more detail available for the patient*” (P24). It is also important to “*Have a plan for when patients don’t turn up*” (P9). Additionally, “*Ensure the student cannot see the assessor on the screen/in the room during the consultation*” (P6). For delayed assessment, “*Ensure there is a recording capability*” (P5), that it is possible “*to view entire consult and be able to stop and start video as needed*” (P17), and the recording is of good quality “*where there is no/poor video it makes assessment difficult”* (P12).

Assessment *via* telehealth requires standardized and clear assessment tools (Subtheme 5.3) that can be utilized by both students and educators to support preparation and transparency with assessment:

“*Having clear assessment guidelines/tools so the student is aware of what is being assessed*” (P6)

There is a need for standardized and validated assessment tools developed for the telehealth setting that captures required adaptation of communication skills:

“*Developing a standard model for the assessment of non-verbal behavior in tele-consultations*” (P10).

Lastly, having confidence in the administration of an assessment tool designed for telehealth were valued by participants:

*“The limiting factor for me is this is the first time using the marking tool so adjusting to that and interpreting it was hardest more than the telehealth aspects”* (P4)

### Ways to improve the online marking experience

A checklist was developed for educators during the planning, preparation and assessment of communication skills using telehealth (see Table 4). Broad categories include student preparation, educator preparation, assessment, and post-assessment.

**TABLE 4:** Checklist items for educators to plan and implement telehealth consultations for undergraduate students.

Category	Item	Check
Student preparation	Ensure students have adequate communication skills and understand how to adapt these to the telehealth setting, e.g., active listening, client led, and counseling	
	
	Prepare students for telehealth setting using resources, videos, briefings, e.g., pre-recorded consults, practice with peers	
	
	Provide opportunities for students to practice skills using telehealth in a safe, supported environment using simulation or reviewing pre-recorded consults	
	
	Support student to build confidence in using technology	
	
	Facilitate the development of telehealth etiquette to improve engagement: appropriate responses in a telehealth consult, how to build and maintain rapport, the use of silence, the pace of the consult	
	
	Familiarize students with learning outcomes and assessment criteria	
	
Educator preparation	Identify learning outcomes of the consultation	
	
	Develop case scenarios that allow students to demonstrate relevant skills	
	
	Design simulation-based learning using evidence-based guidelines	
	
	Identify appropriate assessment tool to assess student skills	
	
	Familiarize self with student assessment tools	
	
	Develop technology skills for platform to be used e.g., waiting room function, recording function	
	
	Check audio and video quality of recordings	
	
	Gain consent for recording from students and simulated participants	
	
Assessment	Allow adequate time for consultation including bringing the client in from the waiting room and explaining the nature of the consultation, e.g., recording, assessor observing	
	
	Turn camera and microphone of supervisor off during consultation	
	
	Provide feedback *via* chat function to student if required to support learning*	
	
Post-assessment	Ensure that there is sufficient time allocated for student debrief	
	
	Make recordings available to students for self-reflection*	
	
	Ensure that there is sufficient time allocated for client debrief with students	
	
	Encourage student reflection	
	
	Arrange for moderation*	

*Relevance of item will depend on whether assessment is formative or summative.

## Discussion

To the authors knowledge, this is the first study to report on the views and perceptions of educators assessing communication skills using telehealth. Five key themes emerged from analyses: non-verbal communication is more difficult to assess using telehealth compared to verbal communication, the need to adapt verbal communication for telehealth, establishment of an optimal telehealth environment, telehealth specific preparation, and assessment *via* telehealth.

Both verbal and non-verbal communication are important for the development of a trusting patient-clinician relationship which is linked to improved patient outcomes (39, 40). Educators in the current study had varied views on the ability to assess communication skills *via* telehealth with some saying it was easy and/or similar to an in-person face-to-face setting whilst others felt some adaptations are required to enable adequate assessment of all communication skills. Verbal communication skills were deemed easier to assess *via* telehealth compared to non-verbal communication skills. These findings support compensatory adaptation theory where those that convey a message through electronic communication adapt to enhance the “naturalness” of the medium (41). Telehealth provides the opportunity for synchronized face-to-face communication which increases its “naturalness,” in other words having a comparable experience to the in-person face-to-face setting. Nevertheless, some adaptation is required when communicating *via* telehealth. Participants in the current study identified it was more challenging to assess rapport building, eye contact, body language, cues, gestures, posture, and use of pause during a consultation. It is recognized that non-verbal communication requires adaptation in a telehealth setting, with motions such as nods, verbal and facial expressions, and body language modified to create a video presence and improve relationship building (2, 21, 40, 42). Furthermore, effective use of pause and silence has been identified by clinicians experienced in the use of telehealth (21) to allow the client to process information and provides an opportunity to express their feelings and values.

Concern has been raised whether clinicians can effectively empathize using computer mediated communication such as teleconferencing (42) which is important for rapport building and ultimately support patient-clinician relationship building (39, 40). Liu and colleagues (43) found doctors had a lower frequency of praise and empathy utterances during a telehealth compared to face-to-face consultations which may influence the patient-clinician relationship. In the current study, educators found it easy to assess empathy and felt it to be similar to in-person assessment, which aligns with media richness theory (44). Thus, telehealth afforded the ability for some student dietitians to portray empathy *via* a videoconference telehealth session which participants could observe and felt confident to assess. It appears that some students in the current study were able to adequately adapt their communication to the telehealth setting while others needed further training in adapting their communication skills. Our results indicate that when communication skills are adapted for the telehealth setting, educators are able to identify both verbal and non-verbal communication skills and feel confident to assess these. On the other hand, if students lack the ability to adapt their communication skills it reduces the confidence of educators to assess these skills.

Despite the rapid uptake of telehealth to deliver healthcare services, literature on telehealth education and training of students and clinicians remains limited (45, 46). Formal training of clinicians using telehealth is recommended with particular focus on verbal and non-verbal communication as well as adapting interpersonal skills to the telehealth environment (45). Participants in the current study suggested students be provided with the opportunity to observe telehealth consultations and to practice utilizing telehealth which is supported by current guidelines (46). The increased complexity of teaching students technology skills and telehealth etiquette was acknowledged as a barrier in the current study. This may have been intensified by the rapid roll-out of telehealth education in the curriculum and on placement as a result of the COVID-19 pandemic (3). Student training on adapting counseling and communication style for the telehealth setting was identified as a need by participants in the current study. Similar to Ferro (2), findings of the current study highlights the need for further research on tailoring nutrition counseling to the telehealth environment to ensure effective computer mediated communication.

A number of recommendations were provided by participants in the current study to both students and educators in general to improve the delivery of nutrition care *via* telehealth. Recommendations align with current telehealth guidelines to clinicians regarding screen etiquette, adapting non-verbal and verbal communication, appropriate preparation, and telehealth environment to optimize the experience (2, 19, 40, 45, 47). There is currently limited guidance for educators responsible for student training and assessment using telehealth and a need for standardized procedures and assessment tools were voiced by participants in the current study. A checklist was developed in the current study from the recommendations provided by participants for educators and students in planning, preparing for, and assessment of telehealth encounters, thereby addressing a gap in the current literature. Recently Henry et al. (48) published a validated checklist to assess interpersonal and communication skills of clinicians using telehealth (48). Our findings align with the items in their checklist and suggest further consideration when applying it for student training.

Three key recommendations from this study are highlighted. Firstly, to foster relationship building between students and patients, students should be discouraged from using verbal scripts. This may not be an issue for experience clinicians, however, can be viewed as a helpful aid for inexperienced students. Students may be tempted to use a script in the telehealth environment as it may be hidden compared to when they are in the same room as the client. In the current study scripts were viewed unfavorably as it did not allow students to tailor their communication to the client’s needs. Telehealth encounters for student training should include nuances of real-life challenges faced by clinicians to better prepare students for the workforce. Engaging clients during a telehealth consultation is one such challenge (49) emphasizing the importance of effective communication skills to build and maintain trusting patient-clinician relationships. Secondly, including moderation was identified as an important consideration for summative assessment of communication skills due to the subjective nature of the assessment of this skill in the current study. Bias on the part of assessors during the assessment of practical skills have been reported in the literature (50). Bacon and colleagues (51) explored the variation in assessors’ judgment of student dietitian’s performance by a video recording of a nutrition consultation, as well as the influence of a group discussion amongst assessors on their judgments. They found no agreement in assessors’ ratings before or after the discussion, although 78% of assessors changed their scores and/or reported a change in confidence levels in assessment. Assessment of procedural activities, albeit still challenging, may be less so than non-tangible aspects like communication skills (50). Nevertheless, assessment of competency in communication and interpersonal skills with video recording is an accepted evaluation method in medical education and other health professions (52). More recently, the use of Zoom teleconferencing software was found to be an appropriate platform to assess various skills including communication skills using Objective Structured Clinical Examination (OSCE) (51) with some recommendations provided for educators when conducting online OSCEs (53). Lastly, to support a deeper level of learning, participants in the current study recommended that educators provide timely feedback, allow time for debriefing and encourage student reflection after the telehealth encounter. These recommendations align with guidelines for the use of virtual simulation programs to assess student clinical competency (54–56).

Limitations to the current study include the use of sections from dietetic interviews rather than the full consultations in order to reduce participant burden. This may have impacted the ability of participants to observe all communication skills. Furthermore, a global communication rating scale was used to facilitate the assessment of communication skills that was not developed for teleconference use. However, similar issues were raised by participants that are included in a recently published checklist for assessment of communication skills using telehealth (48), thus the tool used in the current study did not seem to detract from the marking experience. Only telehealth encounters of pass level students were included, therefore perceptions of participants may be different with the inclusion of borderline or students that failed the encounter was included.

In conclusion, dietetic educators felt it was easier to assess verbal communication skills in telehealth using videoconferencing. Assessment of non-verbal communication skills were challenging and required adaptation and awareness from students to enable educators to observe these skills and allow easier assessment. Despite challenges identified in assessing communication skills, educators in the current study agreed on the importance of including telehealth training in the curriculum to better prepare dietetic students for the future workforce. This study endeavored to shed light on the perceptions of dietetic educators to assess communication skills within the telehealth environment. Our findings reveal a scarcity of recommendations for educators when conducting online assessment of communication skills using telehealth. Further research is warranted to develop best practice guidelines and validated tools for educators when assessing communication skills and competencies using telehealth.

## Data availability statement

The raw data supporting the conclusions of this article will be made available by the authors, without undue reservation.

## Ethics statement

The studies involving human participants were reviewed and approved by the Research and Ethics Committee of Griffith University (No: 2020/881). The patients/participants provided their written informed consent to participate in this study.

## Author contributions

HW and MCO conceptualized the study. HW wrote first draft. All authors contributed to manuscript revision, read, contributed to data analysis, and approved the submitted version.
