# Supplementary material for: Assessment of communication skills using telehealth: considerations for educators
Source: Front Med (Lausanne). 2022 Aug 1;9:841309. doi: 10.3389/fmed.2022.841309 (PMC9377413; doi:10.3389/fmed.2022.841309)
Supplement: Supplementary file 1 [file Table_1.docx]

Supplementary material.

Individual participant characteristics of dietetic educators participating in the study

| **ID** | **Country, State** | **# yr Dietitian for** | **# yr assessing students prior to placement** | **Telehealth experience** |
| --- | --- | --- | --- | --- |
| 1 | NZ | 10 - < 15 years | 3-5years | None |
| 2 | NZ | 7 - < 10 years | > 5 years | Prior to placement |
| 3 | Vic, Aus | > 20 years | > 5 years | Prior to placement |
| 4 | NZ | 15 - < 20 years | > 5 years | Prior to and on placement |
| 5 | Qld, Aus | 10 - < 15 years | > 5 years | Prior to placement |
| 6 | NSW, Aus | > 20 years | > 5 years | On placement |
| 7 | Vic, Aus | > 20 years | 3-5years | None |
| 8 | Vic, Aus | 3 -< 5 years | ≤ 2 years | Prior to placement |
| 9 | NSW, Aus | 10 - < 15 years | > 5 years | Prior to and on placement |
| 10 | NZ | 10 - < 15 years | 3-5years | Prior to placement |
| 11 | NZ | 15 - < 20 years | > 5 years | None |
| 12 | NZ | 10 - < 15 years | 3-5years | Prior to and on placement |
| 13 | NZ | 1 - < 3 years | ≤ 2 years | On placement |
| 14 | NZ | > 20 years | > 5 years | None |
| 15 | Qld, Aus | > 20 years | > 5 years | On placement |
| 16 | Qld, Aus | 10 - < 15 years | > 5 years | None |
| 17 | NZ | > 20 years | > 5 years | None |
| 18 | NZ | 10 - < 15 years | ≤ 2 years | None |
| 19 | NZ | 10 - < 15 years | > 5 years | Prior to placement |
| 20 | Qld, Aus | 15 - < 20 years | > 5 years | None |
| 21 | WA, Aus | 10 - < 15 years | > 5 years | None |
| 22 | SA, Aus | 15 - < 20 years | > 5 years | Prior to placement |
| 23 | NSW, Aus | > 20 years | > 5 years | On placement |
| 24 | NSW, Aus | 15 - < 20 years | > 5 years | On placement |
